# Survival rates of children and young adolescents with CNS tumors improved in the Netherlands since 1990: A population-based study

**DOI:** 10.1093/noajnl/vdab183

**Published:** 2021-12-21

**Authors:** Raoull Hoogendijk, Jasper van der Lugt, Dannis van Vuurden, Leontien Kremer, Pieter Wesseling, Eelco Hoving, Henrike E Karim-Kos

**Affiliations:** 1 Princess Máxima Center for Pediatric Oncology, Utrecht, the Netherlands; 2 Department of Pediatrics, Emma Children’s Hospital/Amsterdam University Medical Center/AMC, Amsterdam, the Netherlands; 3 Department of Pathology, Amsterdam University Medical Center/VUmc, Amsterdam, the Netherlands; 4 Department of Neurosurgery, University Medical Center Utrecht, Utrecht, the Netherlands; 5 Department of Research and Development, Netherlands Comprehensive Cancer Organization (IKNL), Utrecht, The Netherlands

**Keywords:** CNS, epidemiology, incidence, survival, time trends

## Abstract

**Introduction:**

Survival of children with central nervous system (CNS) tumors varies largely between countries. For the Netherlands, detailed population-based estimation of incidence, survival, and mortality of pediatric CNS tumors are lacking but are needed to evaluate progress.

**Methods:**

All CNS tumors diagnosed in patients <18 years during 1990-2017 were selected from the Netherlands Cancer Registry. Other than pilocytic astrocytomas, nonmalignant tumors were included since 2000. Incidence and mortality trends were evaluated by average annual percentage change (AAPC). Changes over time in the five-year observed survival (5-year OS) were evaluated by Poisson regression models adjusted for follow-up time.

**Results:**

Between 1990 and 2017, 2057 children were diagnosed with a malignant CNS tumor and 885 with a pilocytic astrocytoma. During 2000-2017, 695 children were diagnosed with other nonmalignant CNS tumors. Incidence rates of malignant tumors remained stable, while pilocytic astrocytomas and other nonmalignant tumors increased by 2.0% and 2.4% per year, respectively. The 5-year OS rates improved for all groups; however, improvement for malignant tumors was not constant over time. The contribution of malignant tumors located at the optic nerve tumors was 1% in 2000-2009. However, shifting from pilocytic astrocytomas, increased to 6% in 2010-2017, impacting survival outcomes for malignant tumors.

**Conclusion:**

Survival rates of CNS tumors improved over time but were not accompanied by a decreasing mortality rate. The observed temporary survival deterioration for malignant tumors appears to be related to changes in diagnostics and registration practices. Whether differences in treatment regimens contribute to this temporary decline in survival needs to be verified.

**Key Points**

- Survival rates of pediatric CNS tumors improved over time.
- Incidence and mortality rates of malignant CNS tumors remained stable.
- Diagnostic and registration practices of CNS tumors affect survival outcomes.

**Importance of the Study**

Reported survival rates of children with central nervous system (CNS) tumors vary largely across European countries. For the Netherlands, detailed population-based estimation of incidence and survival by CNS tumor subtypes and mortality from CNS tumors in children are lacking. To accurately compare incidence and survival between countries, detailed information on distinct tumor subtypes and harmonized registration criteria are necessary. In the Netherlands, the five-year observed survival (5-year OS) rates improved over time. We observed a slightly decreasing trend in the 5-year OS rates between 2000 and 2009 for malignant CNS tumors. This survival deterioration seems to be partly related to changes in diagnostic and registration practices over time, and importantly, these changes may contribute to OS differences between countries. More detailed future studies will look at potential other underlying reasons (eg, differences in treatment regimens) to explain the periodical deterioration in survival of malignant tumors in 2000-2009 in the Netherlands.

Central nervous system (CNS) tumors have the highest mortality among all childhood cancers and are the most common type of solid tumors diagnosed in children, with an average incidence rate of 35 per million per year in Europe.^1^ Since the 1990s, incidence has increased on average by 1.4% annually in Western Europe.^2^ Moreover, 5-year survival was found to vary largely between European countries and did not improve since the late 1990s.^3^ A recent study, based on 71 population-based cancer registries in 27 countries, surveying the time period of 2000-2007, reported a 5-year survival rate of 46% for pediatric malignant CNS tumors and 96% for nonmalignant CNS tumors in the Netherlands. In contrast, for malignant tumors, an average for the European pool of 57% was reported, with even a 5-year survival rate of 75% observed for Finnish patients.^1^

It is unclear if this variation in survival between countries is real or caused by bias, such as registration bias (ie, changes in inclusion criteria and in coding practices of malignant vs nonmalignant CNS tumors and variation in registration of tumors without microscopic verification) and in completeness of follow-up.^2,3^ Real survival variation could be caused by differences in access to diagnostic facilities (ie, MRI and stereotactic biopsy) and/or the provision of inferior treatment regimens.

In the literature, population-based studies for CNS tumors focusing on the pediatric population are limited. Recently, a Dutch study on trends in childhood cancer incidence (0-17 years) has shown that during 1990-2017 the incidence of CNS tumors, including pilocytic astrocytomas, increased by 1.0% per year, resulting in an average incidence rate of 34 per million person-years in 2010-2017. Pilocytic astrocytomas as a diagnostic subgroup increased by 1.8% per year with a recent average incidence rate of 10 per million person-years.^4^ Previously, survival of CNS tumors had only been studied for the ages 0-14 years in the South of the Netherlands for the 1983-1999 time period. In this study, 5-year survival rates declined from 63% in 1983-1992 to 52% in 1993-1999.^5^ The limitation of these and other international comparative studies is that CNS tumors were analyzed as one entity, while this group consists of a variety of entities differing in incidence and survival outcomes.

The overall aim of this study was to more comprehensively assess the progress made in improving the prognosis for children and young adolescents (0-17 years) with a CNS tumor in the Netherlands by evaluating trends in incidence, survival, and mortality rates since 1990 including detailed analyses regarding CNS tumor subtype and tumor location.

## Methods

### Data Sources

Data were obtained from the Netherlands Cancer Registry (NCR). The NCR, which is managed by the Netherlands Comprehensive Cancer Organization (IKNL), is the only oncological registry in the Netherlands with data on all diagnosed cancer patients with a national coverage since 1989. The NCR has reported a completeness of at least 96% on all patients diagnosed with cancer.^6^ Case notifications are provided through the Nationwide Network and Registry of Histopathology and Cytopathology (PALGA Foundation), and the National Registry of Hospital Discharges.^7^ After notification, relevant information on patient and tumor characteristics is abstracted and coded through retrospective medical records review by trained registrars. Disease data are coded according to the International Classification of Diseases for Oncology (ICD-O)^8–10^ classification relevant at the time of registration.

In line with ICD-O coding rules, CNS tumors were registered including specification of “behavior” (noted as the fifth digit in the morphology code). Behavior of a tumor is the way it acts within the body, and pathologists use a variety of observations to determine this behavior.^8^ For primary CNS tumors behavior codes “/0” (benign), “/1” (uncertain/borderline), or “/3” (malignant) are used. Up until 2000, only malignant CNS tumors (behavior code/3) were registered in the NCR. In concordance with the recommendation of the European Network of Cancer Registries in 1998,^11^ benign and borderline tumors of the CNS (ICD-O-3, behavior codes/0 and/1) were included in the NCR since 2000. Of note in this respect, up until 2000 pilocytic astrocytomas (ICD-O-3 M9421/1) were classified as /3 (malignant) while categorized as /1 (uncertain/borderline) from 2000 onwards.

The Nationwide Population Registries Network holds vital statistics on all residents in the Netherlands and is annually linked to the NCR to gather information on vital status (ie, alive, dead or emigration). Last linkage was at February 1, 2019. Mortality data on patients diagnosed with a CNS tumor [International Classification of Diseases (ICD)-10 code C70-C72)] for the period 1970-2017 were obtained from Statistics Netherlands (CBS).^12^

### Selection of Cases and Definitions

This study included all patients below the age of 18 years registered with an intracranial or intraspinal tumor according to the International Classification of Childhood Cancer (ICCC-3)^13,14^ between 1990 and 2017. We stratified the included patients into 3 main groups of CNS tumors based on their behavior code, that is, malignant tumors (behavior/3, fifth digit morphology code), pilocytic astrocytomas (including pilomyxoid astrocytomas, ICD-O-M-9425/3), and other nonmalignant tumors (behavior/0 and/1, fifth digit morphology code). As pilocytic astrocytomas during the 1990-2017 time period were categorized differently before and after 2000, these were analyzed as a separate group to get more valid estimates for malignant tumors.^15^

We included the ICCC-3 site group “III. CNS and miscellaneous intracranial and intraspinal neoplasms” and the main diagnostic group “Xa Intracranial and intraspinal germ cell tumors.” For “Xa Intracranial and intraspinal germ cell tumors” only tumors with malignant behavior were completely registered and included in this study. As information on nonmalignant tumors were lacking before 2000, for patients diagnosed with a nonmalignant tumor followed by a malignant tumor with an identical histology we only included the malignant tumor.

In total, we selected 3786 malignant and nonmalignant CNS tumor cases. In this selection, cases that did not fulfill the criteria of the International Association of Cancer Registries (IACR) and nonmalignant tumor cases followed by a malignant tumor with an identical histology were deleted. These cases (n = 149) consisted out of ependymomas and choroid plexus tumors (n = 15), astrocytomas and other gliomas (n = 34), other specified intracranial and intraspinal neoplasms (n = 78), unspecified intracranial and intraspinal neoplasms (n = 21), and intracranial and intraspinal germ cell tumors (n = 1).

Fifteen patients were reported to have a second malignant primary tumor, and 2 patients had a third malignant primary tumor with a different histology. These cases were included for a second or third time in further analyses.

The ICCC-3 diagnostic groups “(IIIb) Astrocytomas” and “(IIId) Other gliomas” were merged, as the entities within these groups have a pathological interrelationship. Entities were further grouped according to the 2007 WHO Classification of Tumors of the Central Nervous System^16^ as almost all diagnoses of CNS tumors during the study period were fully histology-based. Tumor location was defined according to the ICD-O-3.^8,9^

A list of the ICCC-3 diagnostic (sub)groups with the corresponding morphological entities and degree of malignancy defined using the WHO CNS grading system for tumors of the CNS^16^ are presented in Supplementary Table S1).

### Statistical Analyses

Characteristics of the study population were described for the 3 main groups of CNS tumors. Results are presented according to 3 time periods: 1990-1999, 2000-2009, and 2010-2017.

Incidence and mortality rates were calculated as the average annual number of cases per million person-years using the annual mid-year population size as obtained from Statistics Netherlands (CBS).^12^ Age-standardized incidence and mortality rates were calculated for the age groups 0-17 and 0-19, respectively. For age standardization, the weights of the Segi world standard population^17^ were used. Age-specific incidence rates were given for the age groups 0, 1-4, 5-9, 10-14, and 15-17. For mortality age-specific rates were provided for the age groups 0, 1-4, 5-9, 10-14, and 15-19. Changes in incidence and mortality rates over time were evaluated by the average annual percentage change (AAPC). The AAPC was derived from a regression line that was fitted to the natural logarithm of the rates using the calendar year as a regressor variable.^18^ Joinpoint regression analysis was performed for incidence and mortality rates to check for trend transitions during the study period. The permutation test was used to determine the number of joinpoints and the null hypothesis assumed that the AAPC was constant throughout the study period. Incidence/mortality rates and microscopic verification percentages were represented in the figures as 3-year moving averages by taking the average of the percentage/rates of each given year and the percentage/rates of either side of it.

Observed survival (OS) was defined as the time from date of diagnosis until death from any cause (ie, event), date of emigration (ie, censored) or to February 1, 2019 (ie, study endpoint), and estimated by the Kaplan-Meier method for 5 years after diagnosis for 3 diagnostic time periods. Although relative survival (RS) is commonly used in epidemiological analyses, OS was used instead of RS as competing causes of death in childhood are rare in developed countries.^19^ Survival changes over time were evaluated by using Poisson regression modeling adjusted for follow-up time (in years)^20^ in which the variable period of diagnosis was entered as a continuous variable in the model (*P*-trend).

Descriptive and survival analyses were performed using R: a language and environment for statistical computing. Analyses on incidence and mortality rates were performed using SAS/STAT software and Joinpoint Regression Program Version 4.8.0.1. A *P*-value <0.05 was considered statistically significant.

## Results

### Patient and Tumor Characteristics

Characteristics of the included CNS tumor patients are presented in Table 1. A total of 3637 children with a diagnosis of a CNS tumor were registered during 1990-2017 in the Netherlands, including 2057 malignant tumors, 885 pilocytic astrocytomas, and 695 other nonmalignant tumors. In 2010-2017, half of the CNS tumors were malignant (51%), and the other half (49%) were classified as nonmalignant, including pilocytic astrocytomas (22%) and other nonmalignant tumors (27%). For malignant tumors, boys were more affected than girls with an B:G ratio of 1.4, and about 60% were diagnosed in children below the age of 10. The most common malignant tumors were “astrocytomas and other gliomas” (41%) and “intracranial and intraspinal embryonal tumors” (30%). Pilocytic astrocytomas slightly more often affected girls than boys (B:G ratio 0.9) and were most commonly diagnosed in children aged 5-9 years (34%). Other nonmalignant tumors had a B:G ratio of 0.8 which is mainly explained by the tumors of the sellar region (N = 280) including n = 89 (31.8%) boys vs n = 191 (68.2%) girls. Nonmalignant tumors were more often diagnosed in young adolescents aged 15-17 years (32%). Overall, these tumors consist mostly out of “other non-specified intracranial and intraspinal tumors” (78%).

**Table 1.** Characteristics of Patients Aged <18 Years Diagnosed With a CNS Tumor in the Netherlands Between 1990 and 2017

	Overall	Malignant Tumors (Excl. Pilocytic Astrocytomas)	Pilocytic Astrocytomas (9421/1, 9421/3, 9425/3^a^)	Nonmalignant Tumors (Excl. Pilocytic Astrocytomas)^b^
	(N = 3637)	(N = 2057)	(N = 885)	(N = 695)
Sex				
Boys	1934 (53.2%)	1198 (58.2%)	429 (48.5%)	307 (44.2%)
Girls	1703 (46.8%)	859 (41.8%)	456 (51.5%)	388 (55.8%)
Age at diagnosis (in years)				
0	183 (5.0%)	129 (6.3%)	29 (3.3%)	25 (3.6%)
1-4	893 (24.6%)	555 (27.0%)	236 (26.7%)	102 (14.7%)
5-9	1039 (28.6%)	601 (29.2%)	299 (33.8%)	139 (20.0%)
10-14	923 (25.4%)	509 (24.7%)	207 (23.4%)	207 (29.8%)
15-17	599 (16.5%)	263 (12.8%)	114 (12.9%)	222 (31.9%)
Period of diagnosis				
1990-1999	932 (25.6%)	688 (33.4%)	244 (27.6%)	
2000-2009	1435 (39.5%)	721 (35.1%)	361 (40.8%)	353 (50.8%)
2010-2017	1270 (34.9%)	648 (31.5%)	280 (31.6%)	342 (49.2%)
ICCC-3 Diagnostic groups				
(IIIa) Ependymomas and choroid plexus tumors	340 (9.3%)	272 (13.2%)		68 (9.8%)
(IIIb) and (IIId) Astrocytomas and other gliomas	1755 (48.3%)	839 (40.8%)	885 (100%)	31 (4.5%)
(IIIc) Intracranial and intraspinal embryonal tumors	616 (16.9%)	616 (29.9%)		
(IIIe) Other specified intracranial and intraspinal neoplasms	587 (16.1%)	43 (2.1%)		544 (78.3%)
(IIIf) Unspecified intracranial and intraspinal neoplasms	213 (5.9%)	161 (7.8%)		52 (7.5%)
(Xa) Intracranial and intraspinal germ cell tumors	126 (3.5%)	126 (6.1%)		
WHO grade				
I	1501 (41.3%)	21 (1.0%)	871 (98.4%)	609 (87.6%)
II	495 (13.6%)	448 (21.8%)	14 (1.6%)	33 (4.7%)
III	275 (7.6%)	275 (13.4%)		
IV	805 (22.1%)	805 (39.1%)		
Germ cell tumors	126 (3.5%)	126 (6.1%)		
Unknown	435 (12.0%)	382 (18.6%)		53 (7.6%)
Microscopic verification				
No	667 (18.3%)	387 (18.8%)	96 (10.8%)	184 (26.5%)
Yes	2970 (81.7%)	1670 (81.2%)	789 (89.2%)	511 (73.5%)

^a^Pilomyxoid astrocytoma n = 14, between 2005 and 2016.

^b^Nonmalignant tumors were registered since the year 2000.

### WHO Grade and Microscopic Verification

When interrogating the malignant tumors and specifying these according to the WHO classification of CNS tumors, it was found that this group consists mostly of high-grade tumors (WHO grade III/IV) with an overall contribution between 1990 and 2017 of 53%. The group of malignant tumors also includes WHO grade I/II tumors (23%) (eg, glioma not otherwise specified (NOS), ependymomas NOS, and low-grade astrocytomas like pleomorphic xanthoastrocytoma). Nonmalignant tumors consist solely out of WHO grade I (88%), WHO grade II (5%), and tumors with an unknown grade (8%). WHO grade II tumors classified as nonmalignant were atypical choroid plexus papilloma (ICD-O-M-9390/1; n = 8), nonmalignant meningioma (ICD-O-M-9538/1, M-9539/1; n = 12), and central neurocytoma (ICD-O-M-9506/1; n = 13) (Supplementary Table S1).

**Figure 1.**
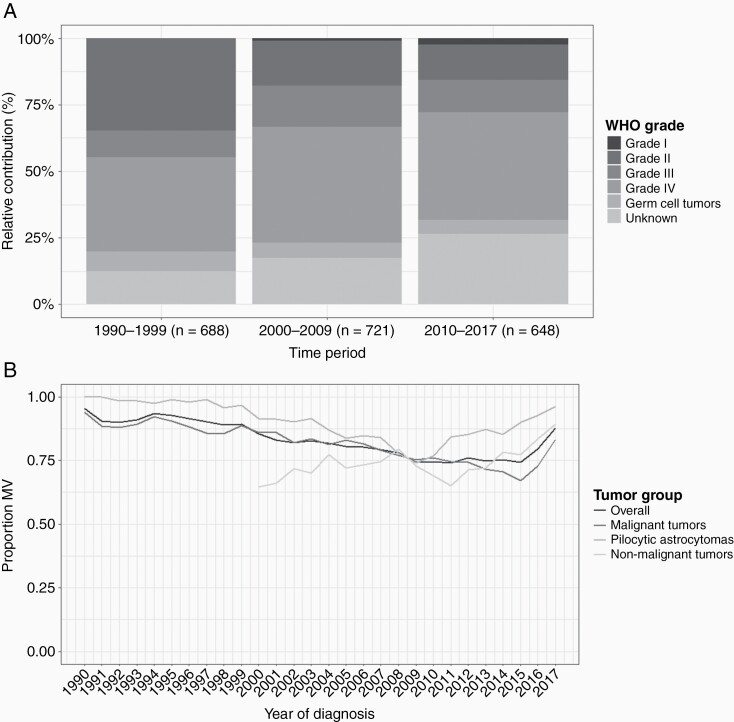
(A) Relative contribution of WHO grade per time period for malignant tumors and (B) the percentage of microscopic verification over time for patients <18 years diagnosed with a CNS tumor in the Netherlands between 1990 and 2017.

Overall we observed an increase in tumors with unknown grading, which is mostly a result of a rise in malignant tumors with an unknown grade (eg, gliomas NOS and malignant unspecified tumors) which increased from 13% in 1990-1999 to 26% in 2010-2017 (Figure 1A). In contrast, for nonmalignant tumors, the contribution of tumors with an unknown grade (ie, gliofibroma and nonmalignant unspecified tumors) remained stable.

Probably related to the increase of malignant tumors with an unknown grade, is an observed decrease in microscopic verification of malignant tumors which steadily decreased from 92% in 2000 to 63% in 2016 (Figure 1B). A comparable pattern of a reduction in microscopic verification was found for pilocytic astrocytomas for the period 2000 (100%) to 2009 (67%). Other nonmalignant tumors remained stable (72%) between 2000 and 2011. In recent years, microscopic verification increased again for all tumor groups, from 73% in 2016 to 88% in 2017. However, the increase of microscopic verification in time differs between groups, as malignant tumors increased after 2016, pilocytic astrocytomas from 2010 and other nonmalignant tumors from 2012 onwards.

### Tumor Location

Table 2 shows the relative distribution of tumor location per time period for malignant tumors, pilocytic astrocytomas, and other nonmalignant tumors, specified by ICCC-3 main diagnostic groups.

**Table 2.** Relative Contribution of Tumor Location per Time Period for Malignant Tumors, Pilocytic Astrocytomas, and Nonmalignant Tumors Specified by ICCC-3 Diagnostic Groups

	(IIIa) Ependymomas and Choroid Plexus Tumor					(IIIb) and (IIId) Astrocytomas and Other Gliomas								(IIIc) Intracranial and Intraspinal Embryonal Tumors			
	Malignant			Nonmalignant		Malignant			Pilocytic Astrocytomas			Nonmalignant			Malignant		
	1990-1999	2000-2009	2010-2017	2000-2009	2010-2017	1990-1999	2000-2009	2010- 2017	1990-1999	2000-2009	2010-2017	2000-2009	2010-2017		1990-1999	2000-2009	2010-2017
	(N = 102)	(N = 104)	(N = 66)	(N = 35)	(N = 33)	(N = 268)	(N = 296)	(N = 275)	(N = 244)	(N = 361)	(N = 280)	(N = 16)	(N = 15)		(N = 190)	(N = 235)	(N = 191)
(C70.0) Cerebral meninges	—	—	—	—	—	—	—	—	—	—	—	—	—	—	—	—	—
(C70.1) Spinal meninges	—	—	—	—	—	—	—	—	—	—	—	—	—	—	—	—	—
(C70.9) Meninges, NOS	—	—	—	—	—	—	—	—	—	—	—	—	—	—	—	—	—
(C71.0) Cerebrum	5 (4.9%)	2 (1.9%)	—	—	—	31 (11.6%)	31 (10.5%)	29 (10.5%)	11 (4.5%)	16 (4.4%)	27 (9.6%)	2 (12.5%)	2 (13.3%)		1 (0.5%)	4 (1.7%)	4 (2.1%)
(C71.1) Frontal lobe	4 (3.9%)	10 (9.6%)	4 (6.1%)	—	—	27 (10.1%)	13 (4.4%)	24 (8.7%)	2 (0.8%)	6 (1.7%)	7 (2.5%)	3 (18.8%)	2 (13.3%)		2 (1.1%)	13 (5.5%)	3 (1.6%)
(C71.2) Temporal lobe	2 (2.0%)	2 (1.9%)	1 (1.5%)	—	—	32 (11.9%)	29 (9.8%)	20 (7.3%)	6 (2.5%)	12 (3.3%)	12 (4.3%)	1 (6.2%)	2 (13.3%)		—	9 (3.8%)	1 (0.5%)
(C71.3) Parietal lobe	4 (3.9%)	4 (3.8%)	1 (1.5%)	—	—	14 (5.2%)	13 (4.4%)	6 (2.2%)	2 (0.8%)	10 (2.8%)	4 (1.4%)	—	—		5 (2.6%)	4 (1.7%)	4 (2.1%)
(C71.4) Occipital lobe	2 (2.0%)	3 (2.9%)	1 (1.5%)	—	—	4 (1.5%)	7 (2.4%)	1 (0.4%)	2 (0.8%)	1 (0.3%)	2 (0.7%)	—	—		2 (1.1%)	1 (0.4%)	2 (1.0%)
(C71.5) Ventricle, NOS	31 (30.4%)	29 (27.9%)	22 (33.3%)	18 (51.4%)	18 (54.5%)	9 (3.4%)	9 (3.0%)	11 (4.0%)	17 (7.0%)	17 (4.7%)	15 (5.4%)	9 (56.2%)	5 (33.3%)		15 (7.9%)	3 (1.3%)	7 (3.7%)
(C71.6) Cerebellum, NOS	20 (19.6%)	7 (6.7%)	9 (13.6%)	—	—	25 (9.3%)	8 (2.7%)	10 (3.6%)	93 (38.1%)	117 (32.4%)	95 (33.9%)	—	—		142 (74.7%)	173 (73.6%)	150 (78.5%)
(C71.7) Brain stem	7 (6.9%)	16 (15.4%)	10 (15.2%)	—	1 (3.0%)	51 (19.0%)	130 (43.9%)	127 (46.2%)	23 (9.4%)	34 (9.4%)	33 (11.8%)	—	—		3 (1.6%)	8 (3.4%)	11 (5.8%)
(C71.8) Overlapping lesion of brain	10 (9.8%)	12 (11.5%)	9 (13.6%)	—	—	47 (17.5%)	25 (8.4%)	25 (9.1%)	15 (6.1%)	13 (3.6%)	12 (4.3%)	1 (6.2%)	1 (6.7%)		10 (5.3%)	13 (5.5%)	6 (3.1%)
(C71.9) Brain, NOS	6 (5.9%)	11 (10.6%)	2 (3.0%)	—	—	15 (5.6%)	13 (4.4%)	4 (1.5%)	31 (12.7%)	34 (9.4%)	14 (5.0%)	—	3 (20.0%)		6 (3.2%)	6 (2.6%)	2 (1.0%)
(C72.0) Spinal cord	11 (10.8%)	8 (7.7%)	6 (9.1%)	14 (40.0%)	13 (39.4%)	9 (3.4%)	15 (5.1%)	8 (2.9%)	2 (0.8%)	12 (3.3%)	13 (4.6%)	—	—		3 (1.6%)	1 (0.4%)	1 (0.5%)
(C72.1) Cauda equina	—	—	1 (1.5%)	3 (8.6%)	1 (3.0%)	—	—	1 (0.4%)	—	—	—	—	—		—	—	—
(C72.3) Optic nerve	—	—	—	—	—	1 (0.4%)	3 (1.0%)	9 (3.3%)	39 (16.0%)	88 (24.4%)	46 (16.4%)	—	—		—	—	—
(C72.5) Cranial nerve, NOS	—	—	—	—	—	—	—	—	—	—	—	—	—		—	—	—
(C72.9) Nervous system, NOS	—	—	—	—	—	3 (1.1%)	—	—	—	1 (0.3%)	—	—	—		1 (0.5%)	—	—
(C75.1) Pituitary gland	—	—	—	—	—	—	—	—	—	—	—	—	—		—	—	—
(C75.2) Craniopharyngeal duct	—	—	—	—	—	—	—	—	—	—	—	—	—		—	—	—
(C75.3) Pineal gland	—	—	—	—	—	—	—	—	1 (0.4%)	—	—	—	—		—	—	—
	(IIIe) Other Specified Intracranial and Intraspinal Neoplasms					(IIIf) Unspecified Intracranial and Intraspinal Neoplasms					(Xa) Intracranial and Intraspinal Germ Cell Tumors						
	Malignant			Nonmalignant		Malignant			Nonmalignant			Malignant					
	1990-1999	2000-2009	2010-2017	2000-2009	2010-2017	1990-1999	2000-2009	2010- 2017	2000-2009	2010-2017		1990-1999	2000-2009	2010-2017			
	(N = 9)	(N = 16)	(N = 18)	(N = 275)	(N = 269)	(N = 68)	(N = 29)	(N = 64)	(N = 27)	(N = 25)		(N = 51)	(N = 41)	(N = 34)			
(C70.0) Cerebral meninges	3 (33.3%)	3 (18.8%)	2 (11.1%)	24 (8.7%)	10 (3.7%)	—	—	—	—	—		—	—	—			
(C70.1) Spinal meninges	—	—	—	3 (1.1%)	4 (1.5%)	—	—	—	—	—		—	—	—			
(C70.9) Meninges, NOS	—	—	—	1 (0.4%)	—	—	—	—	—	—		—	—	—			
(C71.0) Cerebrum	—	2 (12.5%)	—	3 (1.1%)	3 (1.1%)	6 (8.8%)	2 (6.9%)	8 (12.5%)	4 (14.8%)	3 (12.0%)		1 (2.0%)	3 (7.3%)	2 (5.9%)			
(C71.1) Frontal lobe	—	1 (6.2%)	1 (5.6%)	20 (7.3%)	16 (5.9%)	—	4 (13.8%)	2 (3.1%)	4 (14.8%)	—		1 (2.0%)	1 (2.4%)	—			
(C71.2) Temporal lobe	—	1 (6.2%)	2 (11.1%)	45 (16.4%)	49 (18.2%)	—	1 (3.4%)	6 (9.4%)	1 (3.7%)	6 (24.0%)		—	—	—			
(C71.3) Parietal lobe	—	—	2 (11.1%)	7 (2.5%)	11 (4.1%)	—	—	1 (1.6%)	1 (3.7%)	1 (4.0%)		—	—	—			
(C71.4) Occipital lobe	1 (11.1%)	—	—	4 (1.5%)	5 (1.9%)	1 (1.5%)	1 (3.4%)	—	—	—		—	—	—			
(C71.5) Ventricle, NOS	—	—	—	5 (1.8%)	4 (1.5%)	—	1 (3.4%)	3 (4.7%)	1 (3.7%)	3 (12.0%)		12 (23.5%)	8 (19.5%)	7 (20.6%)			
(C71.6) Cerebellum, NOS	—	—	—	3 (1.1%)	3 (1.1%)	3 (4.4%)	2 (6.9%)	3 (4.7%)	1 (3.7%)	2 (8.0%)		1 (2.0%)	—	—			
(C71.7) Brain stem	—	—	—	2 (0.7%)	3 (1.1%)	40 (58.8%)	8 (27.6%)	3 (4.7%)	2 (7.4%)	2 (8.0%)		1 (2.0%)	—	2 (5.9%)			
(C71.8) Overlapping lesion of brain	—	1 (6.2%)	1 (5.6%)	9 (3.3%)	13 (4.8%)	5 (7.4%)	—	1 (1.6%)	2 (7.4%)	1 (4.0%)		2 (3.9%)	2 (4.9%)	2 (5.9%)			
(C71.9) Brain, NOS	—	—	—	2 (0.7%)	3 (1.1%)	4 (5.9%)	5 (17.2%)	7 (10.9%)	1 (3.7%)	2 (8.0%)		9 (17.6%)	6 (14.6%)	1 (2.9%)			
(C72.0) Spinal cord	—	—	—	3 (1.1%)	5 (1.9%)	1 (1.5%)	—	1 (1.6%)	—	—		—	—	—			
(C72.1) Cauda equina	—	—	—	—	—	—	—	—	—	—		—	—	—			
(C72.3) Optic nerve	—	—	—	2 (0.7%)	—	3 (4.4%)	2 (6.9%)	27 (42.2%)	3 (11.1%)	3 (12.0%)		—	—	—			
(C72.5) Cranial nerve, NOS	—	—	—	—	—	1 (1.5%)	—	—	1 (3.7%)	—		—	—	—			
(C72.9) Nervous system, NOS	—	—	—	—	—	—	1 (3.4%)	—	2 (7.4%)	1 (4.0%)		—	1 (2.4%)	—			
(C75.1) Pituitary gland	—	—	—	73 (26.5%)	75 (27.9%)	—	1 (3.4%)	—	2 (7.4%)	—		3 (5.9%)	4 (9.8%)	3 (8.8%)			
(C75.2) Craniopharyngeal duct	—	—	—	67 (24.4%)	64 (23.8%)	—	—	—	—	1 (4.0%)		—	—	—			
(C75.3) Pineal gland	5 (55.6%)	8 (50.0%)	10 (55.6%)	2 (0.7%)	1 (0.4%)	4 (5.9%)	1 (3.4%)	2 (3.1%)	2 (7.4%)	—		21 (41.2%)	16 (39.0%)	17 (50.0%)			

Nonmalignant tumors were registered since the year 2000.

For malignant tumors between 1990-1999 and 2000-2009, tumors localized in the brainstem increased from 15% to 23%. In contrast, during the same periods, overlapping lesion of brain decreased from 11% to 7%. Between 2000-2009 and 2010-2017, brain NOS tumors decreased from 6% to 3% and tumors of the optic nerve increased from 1% to 6%. This increasing contribution of optic nerve tumors to the malignant tumor group was seen in the diagnostic group of “astrocytomas and other gliomas” (+2.3%), and even more pronounced in the “unspecified intracranial and intraspinal tumors” (+35%).

Pilocytic astrocytomas located at the cerebrum increased from 4% to 10% between 2000-2009 and 2010-2017. In contrast, pilocytic astrocytomas with location defined as brain, NOS decreased from 9% to 5% for the same period. Of note, the contribution of pilocytic astrocytomas located at the optic nerve was high in 2000-2009 compared to the other periods (24% vs 16% in 1990-1999 and 2010-2017).

For other nonmalignant tumors, the location cerebral meninges decreased from 7% in 2000-2009 to 3% in 2010-2017, while tumors located at the temporal lobe increased from 13% to 17%, respectively.

### Trends in Incidence

**Figure 2.**
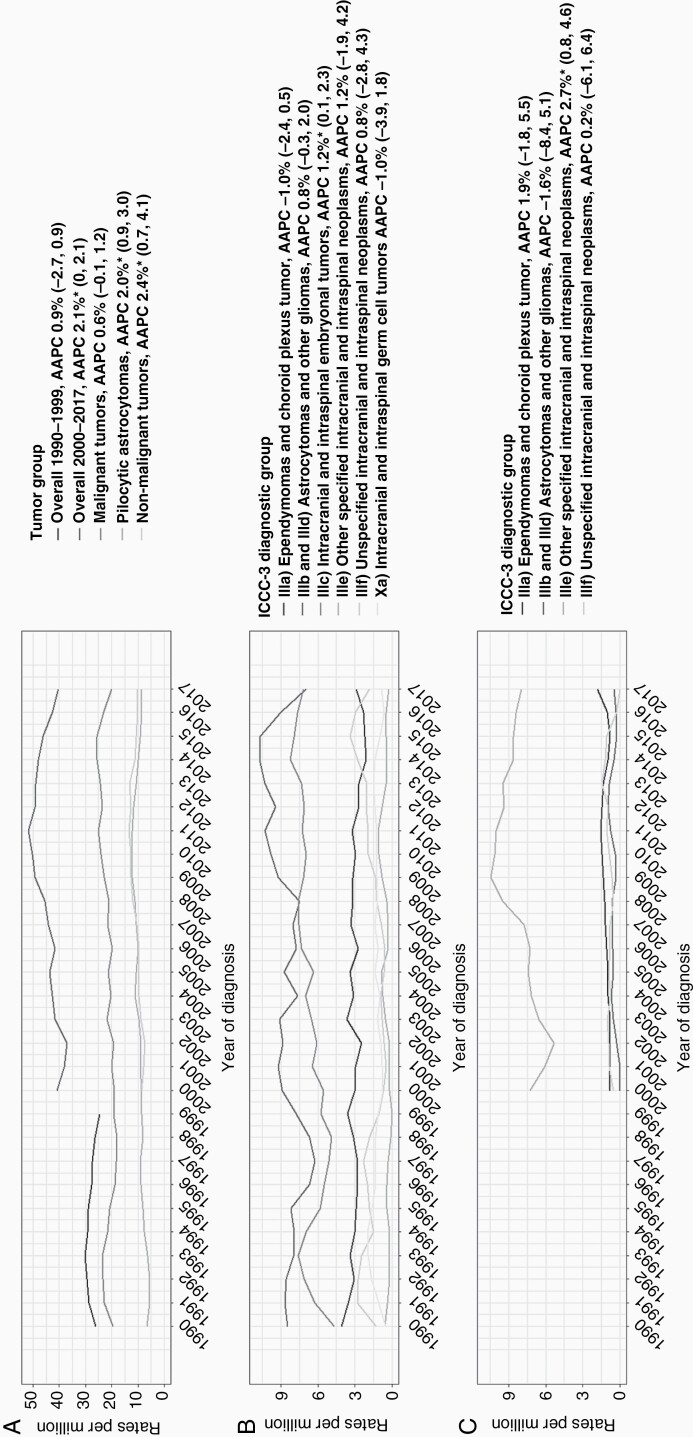
Trends in incidence for (A) overall and main tumor groups and for ICCC-3 diagnostic groups stratified to (B) malignant tumors and (C) nonmalignant tumors. Abbreviations: AAPC, average annual percentage change; ICCC-3, International Classification of Childhood Cancer, Third Edition.

As shown in Figure 2A, incidence for all CNS tumors increased since 2000 with 2.1% per year. During the time period of 1990-2017, incidence rates of malignant tumors seemed to increase slightly by 0.6% per year, but this trend was not significant. Incidence of pilocytic astrocytomas and nonmalignant tumors significantly increased on average by 2% (95% CI 0.9, 3.0) and 2.4% (95% CI 0.7, 4.1) per year, respectively.

Within the group of malignant tumors, a positive trend in incidence rates was found for “intracranial and intraspinal embryonal tumors” which increased by 1.2% (95% CI 0.1, 2.3) per year (Figure 2B). This could mainly be attributed to an increasing incidence rate (4.8% per year, 95% CI 1.5, 8.0) of the subgroup atypical teratoid/rhabdoid tumors (AT/RT). Overall incidence rates of “astrocytomas and other gliomas” remained stable between 1990 and 2017, while subgroups of glioblastoma and variants increased by 5.2% (95% CI 2.5, 7.9) per year and glioma, NOS with 11.6% (95% CI 9.3, 13.9) per year. In contrast, diffuse astrocytomas significantly decreased by 5.9% (95% CI −8.8, −3.1; Supplementary Table S2).

An increase in incidence rates of pilocytic astrocytomas was observed in both boys and girls, and in the age groups 1-4 years (+2.4% per year, 95% CI 0.6, 4.2) and 15-17 years (+4% per year, 95% CI 1.6, 6.5; Supplementary Table S3). The rise in other nonmalignant tumors was most visible in girls (+2.7% per year, 95% CI 0.4, 5.0) and in children aged 5-9 years (+5.7% per year, 95% CI 1.9, 9.6). “Other specified intracranial and intraspinal neoplasms” was the only diagnostic nonmalignant group that significantly increased (+2.7% per year, 95% CI 0.8, 4.6; Figure 2C) which was mainly explained by the increase in the subgroup of neuronal and mixed neuronal-glial tumors (+4.9 per year, 95% CI 2.4, 7.3; Supplementary Table S4). No significant trend transitions were observed for any (sub)group.

### Trends in Survival

**Figure 3.**
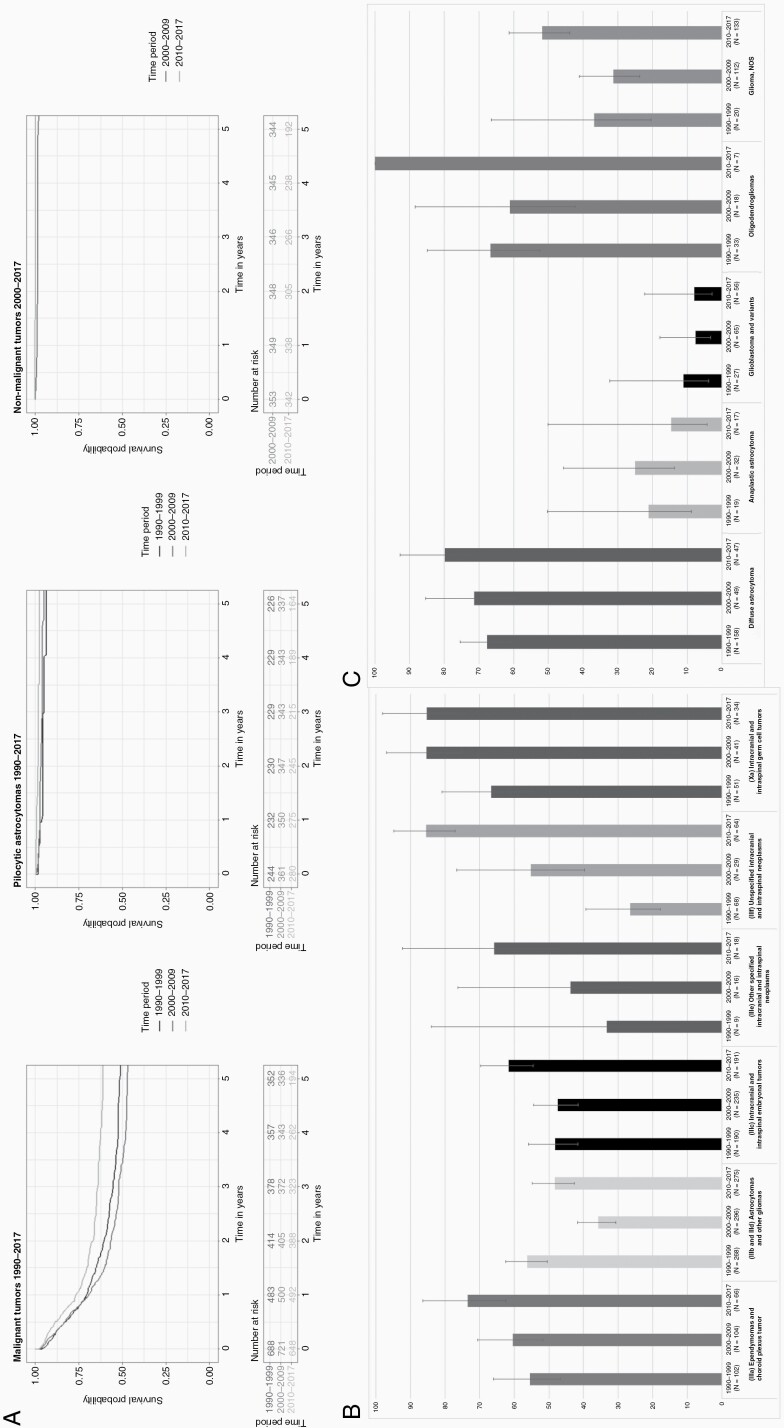
(A) Kaplan-Meier survival curves for each tumor group stratified per time period, (B) five-year observed survival with 95% confidence interval per time period for ICCC-3 diagnostic groups of malignant tumors, and (C) five-year observed survival with 95% confidence interval per time period for malignant astrocytomas and other gliomas. Abbreviation: ICCC-3, International Classification of Childhood Cancer, Third Edition.

Figure 3A shows the 5-year OS rates over time for malignant tumors, pilocytic astrocytomas, and nonmalignant tumors for children and young adolescents in the Netherlands. The largest improvement in the 5-year OS rates was seen for malignant tumors (+10 percent-points, *P*-trend < 0.001) reaching 61% in 2010-2017. However, this improvement was not constant over time, as the 5-year OS rates slightly deteriorated from 51% in the 1990s to 47% in 2000-2009. Five-year OS rates of nonmalignant tumors (+4 percent-points) and pilocytic astrocytomas (+1 percent-points) also improved significantly and came close to 100%.

The survival improvement in malignant tumors was seen in both sexes and in all age groups, except in infants, which showed the poorest prognosis (5-year OS of 41% in 2010-2017). Remarkably, the 5-year OS rates were higher in boys compared to girls, and this difference appeared to enlarge in the latest period (65% vs 56%, respectively). Considering the subgroups, survival improved in all groups, especially in ependymal tumors (+19 percent-points), diffuse astrocytomas (+12 percent-points), and medulloblastomas, variants (+17 percent-points). Five-year OS rates improved significantly over time for all WHO grades with the largest improvement for tumors with unknown grading from 29% in 1999-1999 to 63% in 2010-2017 (*P*-trend < 0.001, Supplementary Table S5).

The observed temporary survival dip between 2000 and 2009 was visible in both sexes, all age groups, except in infants, and only in WHO grade IV tumors. Regarding the ICCC-3 diagnostic groups, the survival dip was mainly seen in “astrocytomas and other gliomas” in which the 5-year OS rate declined from 56% in 1999-1999 to 36% in 2000-2009 followed by an improvement to 61% in 2010-2017 (Figure 3B). This pattern was also seen in malignant glioma, NOS tumors, and glioblastoma and variants (Figure 3C).

For pilocytic astrocytomas and other nonmalignant tumors, an improvement in survival rates was found for both sexes and for all age groups (Supplementary Tables S6 and S7).

### Trends in Mortality

**Figure 4.**
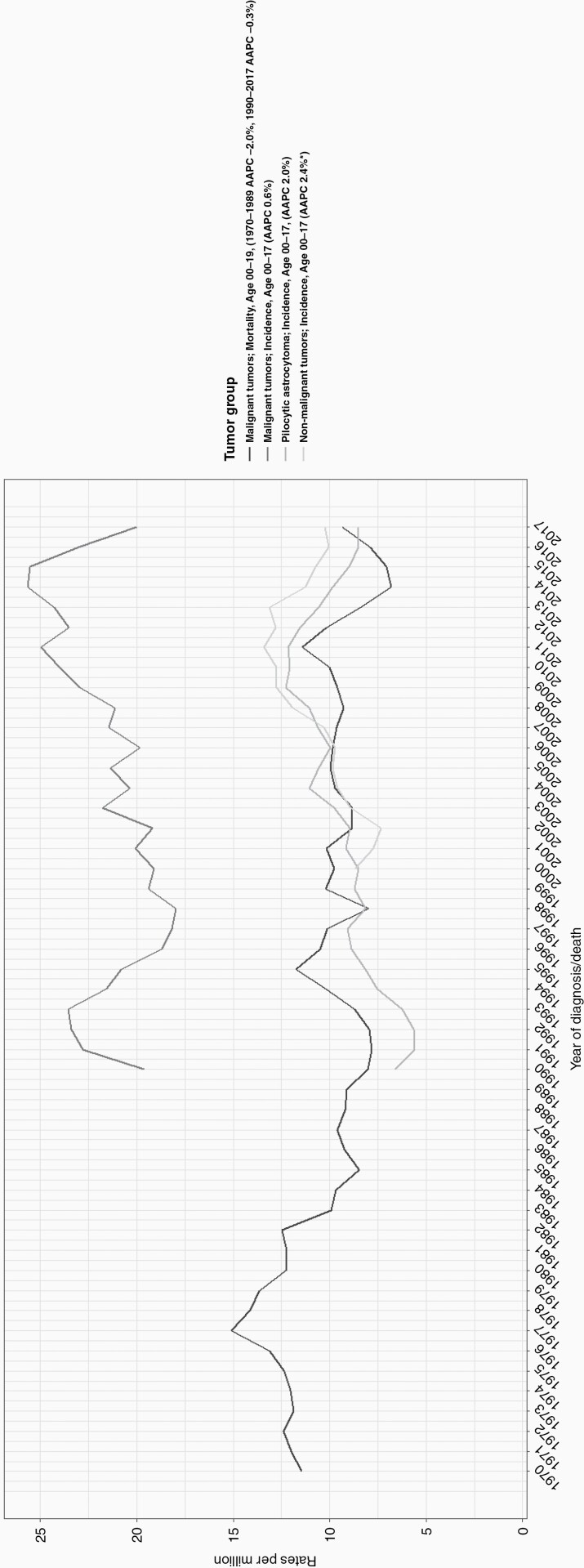
Trends in incidence and mortality of patients diagnosed with a CNS tumor in the Netherlands. Abbreviation: AAPC, average annual percentage change.

The average number of CNS tumor-related deaths in patients aged below 20 years decreased from 56 per year in 1970-1979 to 34 in 2010-2017. Time-trend analyses over 1970-2017 revealed a significant decrease of 0.9% per year (95% CI −1.3, −0.4; Figure 4). In joinpoint analysis, the trend decreased until 1989 by 2% (95% CI −3.7, −0.1) followed by a stable mortality rate with 35 CNS deaths per year for the period 1990-2017. The pattern of a declining mortality rate was seen both in boys and girls, and in the age groups 1-4 years (−1.6% per year, 95% CI −2.5, −0.7) and 15-19 years (−1.2%, per year 95% CI −2.0, −0.3; Supplementary Table S8).

## Discussion

This is the first detailed population-based study on trends in incidence, survival, and mortality rates of CNS tumors among children and adolescents (<18 years) in the Netherlands. Incidence rates of CNS tumors classified as malignant tumors remained stable over time, while incidence rates of pilocytic astrocytomas and other nonmalignant tumors increased. Survival rates for all CNS tumors improved over time, but this improvement was not accompanied by a decrease in mortality rate. For malignant tumors, a temporary decrease in survival rates was observed during 2000-2009.

### Trends in Incidence

A recent European study on incidence trends in malignant CNS tumors in children aged 0-14 years showed a rising trend in western Europe during the period 1991-2010 reaching an incidence rate of 21 per million person-years with an annual change of 0.5% (0.2, 0.8),^2^ while our study found a more or less stable incidence trend at 22 per million person-years for malignant CNS tumors. Our findings are in line with the European average of 22 per million person-years. A contributor to this difference may be the age groups used in these studies, as we also included young adolescents aged 15-17 years. Although not included in the results, we also found a significant positive incidence trend for malignant tumors within the age group 0-14 (+0.7% per year, 95% CI 0.0, 1.3). A recent study reported increased incidence rates in malignant CNS tumors between 1990 and 2017 for the Netherlands.^4^ This study included pilocytic astrocytomas in the malignant tumor group and reported “Intracranial and intraspinal germ cell tumors” separately which explains the reported rise in incidence rates (+1.0%). However, this study also reported an increase in incidence rates of malignant tumors without pilocytic astrocytomas (+0.7%) leading to the conclusion that the stable incidence in our study is due to the inclusion of the “Intracranial and intraspinal germ cell tumors.” We also found an additional 0.2 percent-point rise per year in incidence rates for pilocytic astrocytomas compared to the 1.8% provided by Reedijk et al. This difference is explained by the inclusion of pilomyxoid astrocytomas (ICD-O-M-9425/3) in the pilocytic astrocytoma group in our study.

Within the group of malignant CNS tumors, we observed an increase in incidence rates of “intracranial and intraspinal embryonal tumors” which was especially seen for AT/RT diagnoses. This can partially be explained as AT/RT has only been recognized as a separate entity since 1996^21^ and included in the WHO classification of CNS tumors since 2000.^22^ An additional explanation for this rise of AT/RT is the misclassification of these tumors in the past as PNET or medulloblastoma, as the morphology of AT/RT can be deceivingly similar.^23^ The latter could, however, not explain the observed rise in incidence rate for the diagnostic group of “intracranial and intraspinal embryonal tumors” as more accurate classification of AT/RT would have caused a shift within this group.

In the diagnostic group of “astrocytomas and other gliomas,” the strongest incidence rate increase was found for the subgroup of malignant glioma, NOS. This can be partially explained by the shift of tumors located at the brain stem from the diagnostic group of “unspecified intracranial and intraspinal neoplasms” (tumors located at the brain stem decreased by 31% between 1999-1999 and 2000-2009) toward the group of “astrocytomas and other gliomas” (increased by 25%). At the same time, the percentage of microscopically verified tumors decreased. Possibly, improved imaging techniques enable a more precise clinical diagnosis without the need for microscopic verification. However, the notable increase in the relative contribution of brain stem tumors to the malignant group between 1990-1999 and 2000-2009 (+7.7%) is especially reflected within the subgroup malignant glioma, NOS tumors (+29%). This may also indicate a shift from the nonmalignant tumor group. Unfortunately, nonmalignant tumors were only registered since 2000 making it impossible to test this hypothesis.

A decreasing incidence rate was observed for the subgroup of malignant diffuse astrocytomas which appeared to be associated with the rise in glioblastomas and pilocytic astrocytomas.

Accurate diagnosis of diffuse gliomas (eg, diffuse astrocytomas and glioblastoma) solely based on histology is challenging.^24^ However, increasing use of molecular information during the diagnostic process makes it possible to classify these tumors more accurately which may have led to a shift in diagnoses.

For glioblastomas, this hypothesis is supported by the decrease of CNS WHO grade II tumors (−4.8 per year, 95% CI −6.5, −3.1), and the increase of CNS WHO grade IV tumors (+1.5 per year, 95% CI 0.6, 2.4) within the malignant tumor group. Within the cerebellum, some pilocytic astrocytomas present with a diffuse microscopic appearance which have led to misclassification of these tumors as diffuse astrocytomas in the past.^25^ In addition to improved diagnostics, the increase in incidence rate for pilocytic astrocytomas, and also nonmalignant neuronal and mixed neuronal-glial tumors, may be related to an increase in incidental findings as imaging is more widely used within patient care and research.^26^

### Trends in Survival

Five-year OS rates improved significantly for malignant tumors, pilocytic astrocytomas, and nonmalignant tumors. Improvement in OS rates were most notable for the diagnostic groups of malignant “ependymomas and choroid plexus tumors,” “intracranial and intraspinal embryonal tumors,” and “unspecified intracranial and intraspinal neoplasms.” Contributing factors to the improvement in OS can be found in advancements in diagnostics and treatment. For example, the introduction of molecular subgroups for ependymomas and medulloblastomas led to fewer misdiagnoses followed by more accurate treatment regimens which may have contributed to the increase in OS for these tumors.^27,28^ With these advances, stage migration, that is, “the Will Rogers phenomenon” ^29^ may play a role in the reported improvement in survival. This may be reflected by the increased incidence rate of WHO grade IV tumors and the decreasing incidence rate of WHO grade II tumors within the malignant group. More detailed analyses are needed to provide insight if stage migration is indeed a contributing factor to the improvement in survival.

The improvement in survival rates for malignant tumors was unfortunately not accompanied by a decreasing mortality rate between 1990 and 2017. However, it is worthwhile to keep monitoring the mortality trend as mortality rates seem to decrease from 2011 onward which possibly reflects the improvement in survival for these tumors during 2010-2017.

In this study, we found a temporary dip in the 5-year OS rates of malignant tumors in 2000-2009, with an OS rate of 47%, lower than the European average of 57% found by Gatta et al.^1^ This was especially worse for WHO grade III/IV tumors in the Netherlands (5-year OS 40%, 95% CI 38-46). However, in our most recent period, 2010-2017, the overall 5-year OS rate improved toward 61%: WHO grade III and IV tumors reached a 5-year OS rate of 48% and 50%, respectively. These results are comparable with the European pool for grade III/IV tumors (5-year OS 49%, 95% CI 48-51) reported by Gatta et al.^1^ However, an increase in the 5-year OS rate of ≈10% for WHO grade III and IV tumors cannot fully be explained by improved diagnostics and treatment advocating for other underlying mechanisms.

Our results show that the main contributing diagnostic group to the dip in survival rates for malignant tumors is the “astrocytomas and other gliomas” group. Specifically, the subgroup of glioma, NOS tumors appears to play an important role. For 2000-2009, these tumors (n = 112) had a 5-year OS rate of 31% (95% CI 24-41) compared to 52% (95% CI 44-61) in 2010-2017 (n = 133). This might be explained by the fact that in the 2000-2009 time period, brain stem tumors with a dismal outcome (eg, diffuse midline gliomas) shifted from the diagnostic group of malignant “unspecified intracranial and intraspinal neoplasms” to the “astrocytomas and other gliomas” group, specifically the subgroup glioma, NOS. This consequently led to an improvement in the 5-year OS rate for the malignant unspecified “intracranial and intraspinal neoplasms” group. This shift could be a contributing factor to the dismal outcomes of “astrocytomas and other gliomas” between 2000 and 2009 and may be the effect of advancements in the field of neuroradiological imaging. However, it cannot explain the overall decrease in survival rate between 2000 and 2009 of malignant tumors as this shift occurred within the malignant group.

Also shown in our results is a shift of optic nerve tumors from the pilocytic astrocytomas to the malignant tumor group which appears to be related to a temporary change in registration practices. This shift partially explains the improvement in survival rate for malignant tumors as after excluding optic nerve tumors from the malignant tumor group, the 5-year OS rate remained stable for the periods 1999-1999 and 2000-2009 but decreased for the period 2010-2017 with 2% to 59%. In contrast, including optic nerve tumors from the pilocytic astrocytoma group to the malignant CNS tumors for the period 2000-2009 added more than 5 percent-point to the 5-year OS rate resulting in a 5-year OS rate of 52% (95% CI 49-56) without having an impact on the survival rate of pilocytic astrocytomas (5-year OS 95%, 95% CI 92-97). This shows that survival differences may be contaminated by diagnostic and registration practices and stresses the importance of international harmonization in registration criteria for non-microscopically verified tumors, and detailed comparison of survival between countries for CNS tumors.

### Strengths and Limitations

The strength of our study is its population-based nature and the NCR not having age or hospital limits. In a previous study, we have linked the NCR with the Registry of the Dutch Childhood Oncology Group, which showed that 21% of children and adolescents with a CNS tumor below the age of 18 years were unknown in pediatric oncology centers.^30^ A limitation of our study is that we could only include other nonmalignant tumors since 2000 due to registration practices of the NCR. For that reason, we needed to classify tumors according to behavior which is not in line with clinical practice where WHO grading is used. However, to increase the clinical relevance we have studied time trends for CNS tumors according to the WHO 2007 classification. From another standpoint making use of the WHO 2007 classification can also be seen as a limitation as more recent histomolecular-based classifications of CNS tumors are already available and used in the clinic.^31–33^ Given the fast-changing insights in tumor biology and the rapidly succeeding CNS tumor classifications using the most up-to-date CNS classifications for historical cancer registry data remains challenging. It is therefore worthwhile to explore possibilities how cancer registries can incorporate these developments to increase the value and clinical usefulness of historical data.

Lastly, this article provides detailed information on tumor location and microscopic verification which provides insight into diagnostics and tumor classification over time which is needed for accurate international comparison of CNS tumors.

## Conclusion

This is the first population-based study which gives a detailed overview on trends in incidence, survival, and mortality rates among children and adolescents (<18 years) with CNS tumors in the Netherlands. Incidence rates of malignant tumors remained stable over time, while incidence rates of pilocytic astrocytomas and other nonmalignant tumors increased. Survival rates improved over time for all CNS tumors. However, a temporary decrease in the 5-year OS rate was found for malignant tumors in 2000-2009. Registration and diagnostic practices changed over time which may have influenced our survival outcomes. This study serves as a first step for future research as additional studies are needed to further explain the underlying reasons (eg, differences in treatment regimens) for the periodical deterioration in survival of malignant tumors between 2000 and 2009. In addition, this study stresses the importance of harmonization in registration criteria for non-microscopically verified tumors and international comparative studies to analyze CNS tumors on a detailed clinical level as clustering tumors will lead to inaccurate results and may contaminate survival outcomes.

## Supplementary Material

vdab183_suppl_Supplementary_Table_S1

vdab183_suppl_Supplementary_Table_S2

vdab183_suppl_Supplementary_Table_S3

vdab183_suppl_Supplementary_Table_S4

vdab183_suppl_Supplementary_Table_S5

vdab183_suppl_Supplementary_Table_S6

vdab183_suppl_Supplementary_Table_S7

vdab183_suppl_Supplementary_Table_S8

## Notes

**Authorship statement**. Study design: R.H., H.K.K., J.v.d.L., and D.v.V. Data collection and analyses: Netherlands Comprehensive Cancer Organization (Data collection), R.H. and H.K.K. (analyses). Data interpretation and writing of the manuscript: R.H. and H.K.K. Revision of the manuscript and final approval of the manuscript: all authors.
