# Supplementary material for: Survival rates of children and young adolescents with CNS tumors improved in the Netherlands since 1990: A population-based study
Source: Neurooncol Adv. 2021 Dec 21;4(1):vdab183. doi: 10.1093/noajnl/vdab183 (PMC9113443; doi:10.1093/noajnl/vdab183)
Supplement: vdab183_suppl_Supplementary_Table_S1 [file vdab183_suppl_supplementary_table_s1.docx]

Table S1. ICCC-3 subgroups with corresponding morphological entities, degree of malignancy defined using the WHO CNS grading system for tumors of the central nervous system, and tumor group according to the behavior code

|  |  | ***WHO Grade*** | ***Tumor Group*** |
| --- | --- | --- | --- |
| ***1. (IIIa) Ependymomas and choroid plexus tumor*** |  |  |  |
| *1a.Ependymal tumors* |  |  |  |
| 1a. subependymoma (9383/1) |  | I | NM |
| 1a. myxopapillary ependymoma (9394/1) |  | I | NM |
| 1a. anaplastic ependymoma (9392/3) |  | III | M |
| 1a. ependymoma, other and NOS (9391/3,9393/3) |  | II-III | M |
|  |  |  |  |
| *1b Choroid plexus tumors* |  |  |  |
| 1b. choroid plexus papilloma (9390/0) |  | I | NM |
| 1b. atypical choroid plexus papilloma (9390/1) |  | II | NM |
| 1b. choroid plexus carcinoma (9390/3) |  | III | M |
|  |  |  |  |
| ***2. (IIIb and IIId) Astrocytomas and other gliomas*** |  |  |  |
| *2a.Pilocytic astrocytomas* |  |  |  |
| 2a. pilocytic astrocytoma (9421/1) |  | I | PA |
| 2a. pilomyxoid astrocytoma (9425/3) |  | II | PA |
|  |  |  |  |
| *2b. Diffuse astrocytoma* |  |  |  |
| 2b. low grade astrocytoma (9410/3, 9411/3, 9420/3, 9400/3) | | II | M |
|  |  |  |  |
| *2c. Anaplastic astrocytoma* |  |  |  |
| 2c. anaplastic astrocytoma (9401/3) |  | III | M |
|  |  |  |  |
| *2d. Unique astroctyoma variants* |  |  |  |
| 2d. gliomatosi cerebri (9381/3) |  | III | M |
| 2d. subependymal giant cell astrocytoma (9384/1) |  | I | NM |
| 2d. pleomorphic xanthoastrocytoma (9424/3) |  | II | M |
|  |  |  |  |
| *2e. Glioblastoma* |  |  |  |
| 2e. glioblastoma and variants (9440-9442/3, 9385/3) |  | IV | M |
| 2e. gliofibroma (9442/1) |  | unknown | NM |
|  |  |  |  |
| *2f. Oligodendrogliomas* |  |  |  |
| 2f. oligodendroglioma (9450/3) |  | II | M |
| 2f. oligodendroglioma, anaplastic (9451/3) |  | III | M |
|  |  |  |  |
| *2g. Oligoastrocytic tumors* |  |  |  |
| 2g. oligoastrocytic tumors, anaplastic (9382/3) |  | II, III | M |
|  |  |  |  |
| *2h. Glioma, NOS* |  |  |  |
| 2h. glioma NOS (incl. Optic nerve) (9380/3) |  | I-IV,  unknown | M |
| 2h. angiocentric glioma (9431/1) |  | I | NM |
|  |  |  |  |
|  |  |  |  |
| ***3. (IIIc) Intracranial and intraspinal embryonal tumors*** | |  |  |
| 3. medulloblastoma, variants (9470/3, 9472/3, 9475/3, 9478/3, 9501/3) |  | IV | M |
| 3. desmoplastic/nodular medulloblastoma (9471/3) |  | IV | M |
| 3. PNET, variants (9473/3) |  | IV | M |
| 3. medulloblastoma large cell/anaplastic (9474/3) |  | IV | M |
| 3. Atypical teratoid/rhabdoid tumour (9508/3) |  | IV | M |
|  |  |  |  |
| ***4. (IIIe) Other specified intracranial and intraspinal neoplasms*** | |  |  |
| *4a. Neuronal and mixed neuronal-glial tumors* |  |  |  |
| 4a. dysembryoplastic neuroepithelial tumour (9413/0) |  | I | NM |
| 4a. gangliocytoma, ganglioglioma (9492/0, 9505/1) |  | I | NM |
| 4a. ganglioglioma, anaplastic (9505/3) |  | III | M |
| 4a. central neurocytoma (9506/1) |  | II | NM |
| 4a. papillary glioneuronal tumour (9509/1) |  | I | NM |
| 4a. desmoplastic infantile astrocytoma (9412/1) |  | I | NM |
|  |  |  |  |
| *4b. tumors of the pineal region* |  |  |  |
| 4b. pineocytoma (9361/1) |  | I | NM |
| 4b. pineoblastoma (9362/3) |  | IV | M |
| 4b. papillary tumour of the pineal region (9395/3) |  | II-III | M |
|  |  |  |  |
| *4c. Meningiomas* |  |  |  |
| 4c. meningioma, non-malignant (9530/0, 9531/0, 9532/0, 9533/0, 9534/0, 9537/0, 9538/1, 9539/1) |  | I-II | NM |
| 4c. meningioma, malignant (9530/3, 9538/3, 9539/3) |  | III | M |
|  |  |  |  |
| *4d. tumors of the sellar region* |  |  |  |
| 4d. tumors of the pituitary (8140/0, 8270/0, 8271/0, 8272/0, 8280/0, 8300/0, 9432/1) |  | I | NM |
| 4d. craniopharyngioma (9350/1, 9351/1, 9352/1) |  | I | NM |
|  |  |  |  |
| ***5. (IIIf) Unspecified intracranial and intraspinal neoplasms*** | |  |  |
| 5. non-malignant unspecified (8000/0, 8000/1) |  | unknown | NM |
| 5. malignant unspecified (8000/3, 8002/3) |  | unknown | M |
|  |  |  |  |
| ***6. (Xa) Intracranial and intraspinal germ cell tumors*** |  |  |  |
| 6. germinoma (9060/3, 9061/3, 9064/3) |  | - | - |
| 6. yolk sac tumour (9071/3) |  | - | - |
| 6. teratoma, malignant/immature (9080/3) |  | - | - |
| 6. mixed forms (9085/3, 9101/3) |  | - | - |

***Abbreviations: M, Malignant; NM, Non-malignant; PA, Pilocytic Astrocytomas***
