# Supplementary material for: Survival rates of children and young adolescents with CNS tumors improved in the Netherlands since 1990: A population-based study
Source: Neurooncol Adv. 2021 Dec 21;4(1):vdab183. doi: 10.1093/noajnl/vdab183 (PMC9113443; doi:10.1093/noajnl/vdab183)
Supplement: vdab183_suppl_Supplementary_Table_S2 [file vdab183_suppl_supplementary_table_s2.docx]

Table S2 Average number of new malignant CNS tumor cases (excl. pilocytic astrocytomas) per year, average incidence rate per million person-years and AAPC over time for gender, age, ICCC-3 subgroups and WHO CNS grade in children and young adolescents (aged 0-17 years) in the Netherlands

|  | **Malignant tumors (excl. pilocytic astrocytomas)** | | | | | | | | | |
| --- | --- | --- | --- | --- | --- | --- | --- | --- | --- | --- |
|  | **Total number of cases** | **Average number of new cases per year** | | | | **Average incidence rate per million person-years** | | | | **AAPC % (95% CI)** |
|  | **1990-2017** | **1990-2017** | **1990-99** | **2000-09** | **2010-17** | **1990-17** | **1990-99** | **2000-09** | **2010-17** | **1990-2017** |
| **Total** | 2057 | 73 | 69 | 72 | 81 | 21,6 | 20,6 | 20,6 | 24,3 | 0.6 (-0.1, 1.2) |
| **Gender** |  |  |  |  |  |  |  |  |  |  |
| Boys | 1198 | 43 | 39 | 43 | 47 | 12,5 | 11,7 | 12,2 | 13,9 | 0.7 (-0.3, 1.6) |
| Girls | 859 | 31 | 30 | 29 | 34 | 9,1 | 8,9 | 8,4 | 10,3 | 0.5 (-0.4, 1.3) |
| **Age at diagnosis (in years)** |  |  |  |  |  |  |  |  |  |  |
| 0 | 129 | 5 | 4 | 4 | 6 | 24,6 | 21 | 21,6 | 32,6 | 2.0 (-0.5, 4.6) |
| 1-4 | 555 | 20 | 20 | 19 | 21 | 25,9 | 25,1 | 24,1 | 29,2 | 0.5 (-0.5, 1.5) |
| 5-9 | 601 | 21 | 19 | 23 | 23 | 22,3 | 19,9 | 23 | 24,6 | 0.8 (-0.6, 2.2) |
| 10-14 | 509 | 18 | 17 | 18 | 20 | 18,8 | 18,3 | 18,2 | 20,3 | 0.2 (-1.1, 1.5) |
| 15-17 | 263 | 9 | 10 | 8 | 11 | 16,1 | 17,7 | 13,4 | 17,6 | -0.2 (-2.0, 1.5) |
| **ICCC-3 main diagnostic groups** |  |  |  |  |  |  |  |  |  |  |
| *(IIIa) Ependymomas and choroid plexus tumor* | 272 | 10 | 10 | 10 | 8 | *3* | *3,2* | *3,1* | *2,6* | *-1.0 (-2.4, 0.5)* |
| Ependymal tumors | 252 | 9 | 10 | 10 | 8 | 2,7 | 2,9 | 2,9 | 2,3 | -1.1 (-2.7, 0.5) |
| Choroid plexus tumors | 20 | 1 | 1 | 1 | 1 | 0,2 | 0,2 | 0,2 | 0,3 | 1.3 (1.8, 4.4) |
| *(IIIb and IIId) Astrocytomas and other gliomas* | 839 | 30 | 27 | 30 | 34 | *8,6* | *7,9* | *8,2* | *9,9* | *0.8 (-0.3, 2.0)* |
| Diffuse astrocytoma | 254 | 9 | 16 | 5 | 6 | 2,6 | 4,7 | 1,4 | 1,7 | **-5.9 (-8.8,-3.1)** |
| Anaplastic astrocytoma | 68 | 2 | 2 | 3 | 2 | 0,7 | 0,6 | 0,9 | 0,6 | -0.4 (-3.6, 2.8) |
| Unique astroctyoma variants | 20 | 1 | 0 | 1 | 1 | 0,2 | 0,1 | 0,2 | 0,3 | *NA* |
| Glioblastoma and variants | 148 | 5 | 3 | 7 | 7 | 1,5 | 0,8 | 1,8 | 1,9 | **5.2 (2.5, 7.9)** |
| Oligodendrogliomas | 58 | 2 | 3 | 2 | 1 | 0,6 | 0,9 | 0,5 | 0,3 | -2.5 (-5.2, 0.3) |
| Oligoastrocytic tumors | 26 | 1 | 1 | 1 | 1 | 0,3 | 0,2 | 0,3 | 0,2 | -1.5 (-4.3, 1.4) |
| Glioma , NOS | 265 | 9 | 2 | 11 | 17 | 2,8 | 0,6 | 3,2 | 5 | **11.6 (9.3, 13.9)** |
| *(IIIc) Intracranial and intraspinal embryonal tumors* | 616 | 22 | 19 | 24 | 24 | *6,7* | *5,8* | *6,9* | *7,5* | ***1.2 (0.1, 2.3)*** |
| medulloblastoma, variants | 377 | 13 | 12 | 14 | 14 | 4 | 3,8 | 4,1 | 4,1 | 0.3 (-1.2, 1.8) |
| desmoplastic/nodular medulloblastoma | 68 | 2 | 2 | 2 | 4 | 0,7 | 0,5 | 0,6 | 1,2 | 2.4 (-0.2, 5.0) |
| PNET, variants | 106 | 4 | 5 | 4 | 2 | 1,2 | 1,4 | 1,3 | 0,7 | -2.8 (-6.1, 0.5) |
| medulloblastoma large cell/anaplastic | 14 | 1 | 0 | 1 | 1 | 0,2 | 0 | 0,1 | 0,3 | *NA* |
| Atypical teratoid/rhabdoid tumors | 51 | 2 | 1 | 2 | 3 | 0,6 | 0,2 | 0,7 | 1,1 | **4.8 (1.5, 8.0)** |
| *(IIIe) Other specified intracranial and intraspinal neoplasms* | 43 | 2 | 1 | 2 | 2 | *0,4* | *0,3* | *0,4* | *0,7* | *1.2 (-1.9, 4.2)* |
| Neuronal and mixed neuronal-glial tumors | 12 | <1 | <1 | 1 | 1 | 0,1 | 0 | 0,1 | 0,2 | *NA* |
| tumors of the pineal region | 23 | 1 | 1 | 1 | 1 | 0,2 | 0,2 | 0,2 | 0,4 | 2.0 (-0.5, 4.5) |
| Meningiomas | 8 | <1 | <1 | <1 | <1 | 0,1 | 0,1 | 0,1 | 0,1 | *NA* |
| tumors of the sellar region | - | - | - | - | - | - | - | - | - | *NA* |
| *(IIIf) Unspecified intracranial and intraspinal neoplasms* | 161 | 6 | 7 | 3 | 8 | *1,7* | *2,1* | *0,8* | *2,4* | *0.8 (-2.8, 4.3)* |
| *(Xa) Intracranial and intraspinal germ cell tumors* | 126 | 5 | 5 | 4 | 4 | *1,2* | *1,4* | *1,1* | *1,1* | *-1.0 (-3.9, 1.8)* |
| ***WHO CNS Grade*** |  |  |  |  |  |  |  |  |  |  |
| *WHO grade I* | *21* | 1 | 0 | 1 | 2 | *0,2* | *0* | *0,2* | *0,5* | *NA* |
| *WHO grade II* | *448* | 16 | 24 | 12 | 11 | *4,7* | *7,1* | *3,5* | *3,1* | ***-4.8 (-6.5, -3.1)*** |
| *WHO grade III* | *275* | 10 | 8 | 12 | 10 | *2,9* | *2,2* | *3,4* | *3,2* | *1.9 (-0.2, 4.1)* |
| *WHO grade IV* | *805* | 29 | 24 | 31 | 32 | *8,6* | *7,2* | *8,9* | *9,9* | ***1.5 (0.6, 2.4)*** |
| Unknown grade | *382* | 14 | 9 | 13 | 21 | *4,1* | *2,6* | *3,5* | *6,4* | ***4.8 (3.1, 6.5)*** |

**Abbrevations: NA, Not Assessed -** estimation of a reliable average annual percentage change was not possible because of N = 0 in ≥1 incidence year(s)**; AAPC, Average Annual Percentage Change; 95%CI, 95 percent Confidence Interval**
