# Supplementary material for: Survival rates of children and young adolescents with CNS tumors improved in the Netherlands since 1990: A population-based study
Source: Neurooncol Adv. 2021 Dec 21;4(1):vdab183. doi: 10.1093/noajnl/vdab183 (PMC9113443; doi:10.1093/noajnl/vdab183)
Supplement: vdab183_suppl_Supplementary_Table_S3 [file vdab183_suppl_supplementary_table_s3.docx]

Table S3 Average number of new pilocytic astrocytoma cases per year, average incidence rate per million person-years and AAPC over time for gender and age in children and young adolescents (aged 0-17 years) in the Netherlands

|  | **Pilocytic astrocytomas (9421/1, 9421/3, 9425/3^a^)** | | | | | | | | | |
| --- | --- | --- | --- | --- | --- | --- | --- | --- | --- | --- |
|  | **Total number of cases** | **Average number of new cases per year** | | | | **Average incidence rate**  **per million person-years** | | | | **AAPC % (95% CI)** |
|  |  |  |  |  |  |  |  |  |  |  |
|  | **1990-2017** | **1990-2017** | **1990-99** | **2000-09** | **2010-17** | **1990-2017** | **1990-99** | **2000-09** | **2010-17** | **1990-2017** |
| **Total** | 885 | 32 | 24 | 36 | 35 | 9,2 | 7,3 | 10,3 | 10,4 | **2.0 (0.9, 3.0)** |
| **Gender** |  |  |  |  |  |  |  |  |  |  |
| Boys | 429 | 15 | 12 | 17 | 17 | 4,5 | 3,7 | 4,7 | 5,2 | **1.8 (0.5, 3.0)** |
| Girls | 456 | 16 | 12 | 19 | 18 | 4,8 | 3,5 | 5,6 | 5,3 | **2.1 (0.4, 3.8)** |
| **Age at diagnosis (in years)** |  |  |  |  |  |  |  |  |  |  |
| 0 | 29 | 1 | <1 | 1 | 2 | 5,6 | 2,1 | 5,7 | 9,8 | *NA* |
| 1-4 | 236 | 8 | 7 | 9 | 10 | 11,1 | 8,8 | 11,6 | 13,3 | **2.4 (0.6, 4.2)** |
| 5-9 | 299 | 11 | 8 | 14 | 10 | 11,0 | 8,6 | 13,8 | 10,5 | 1.1 (-0.6, 2.8) |
| 10-14 | 207 | 7 | 6 | 9 | 7 | 7,6 | 6,9 | 8,9 | 7,0 | 0.6 (-1.0, 2.2) |
| 15-17 | 114 | 4 | 3 | 3 | 7 | 7,0 | 5,0 | 5,6 | 11,0 | **4.0 (1.6, 6.5)** |

^a^ Pilomyxoid astrocytoma n=14, between 2005 and 2016

**Abbrevations: NA, Not Assessed -** estimation of a reliable average annual percentage change was not possible because of N = 0 in ≥1 incidence year(s)**; AAPC, Average Annual Percentage Change; 95%CI, 95 percent Confidence Interval**
