# Supplementary material for: Survival rates of children and young adolescents with CNS tumors improved in the Netherlands since 1990: A population-based study
Source: Neurooncol Adv. 2021 Dec 21;4(1):vdab183. doi: 10.1093/noajnl/vdab183 (PMC9113443; doi:10.1093/noajnl/vdab183)
Supplement: vdab183_suppl_Supplementary_Table_S4 [file vdab183_suppl_supplementary_table_s4.docx]

Table S4 Average number of new non-malignant CNS tumor cases (excl. pilocytic astrocytomas) per year, average incidence rate per million person-years and AAPC over time for gender, age, ICCC-3 subgroups and WHO CNS grade in children and young adolescents (aged 0-17 years) in the Netherlands

|  | **Non-Malignant tumors (excl. pilocytic astrocytomas)** | | | | | | | |
| --- | --- | --- | --- | --- | --- | --- | --- | --- |
|  | **Total number of cases** | **Average number of new cases**  **per year** | | | **Average incidence rate per million person-years** | | | **AAPC % (95% CI)** |
|  | **2000-2017** | **2000-2017** | **2000-09** | **2010-17** | **2000-2017** | **2000-09** | **2010-17** | **2000-2017** |
| **Total** | 695 | 39 | 35 | 43 | 10,6 | 9,6 | 11,8 | **2.4 (0.7, 4.1)** |
| **Gender** |  |  |  |  |  |  |  |  |
| Boys | 307 | 17 | 16 | 18 | 4,7 | 4,5 | 5 | 2.0 (-0.4, 4.4) |
| Girls | 388 | 22 | 19 | 25 | 5,9 | 5,1 | 6,8 | **2.7 (0.4, 5.0)** |
| **Age at diagnosis (in years)** |  |  |  |  |  |  |  |  |
| 0 | 25 | 1 | 2 | 1 | 7,5 | 7,8 | 7,1 | 0.4 (-3.6, 4.4) |
| 1-4 | 102 | 6 | 5 | 6 | 7,5 | 6,8 | 8,3 | 2.6 (0.1, 5.1) |
| 5-9 | 139 | 8 | 7 | 9 | 7,9 | 6,7 | 9,5 | **5.7 (1.9, 9.6)** |
| 10-14 | 207 | 12 | 11 | 12 | 11,6 | 11,4 | 11,8 | 2.1 (-2.2, 6.3) |
| 15-17 | 222 | 12 | 10 | 15 | 20,8 | 17,7 | 24,6 | 1.9 (-1.6, 5.4) |
| **ICCC-3 main diagnostic groups** |  |  |  |  |  |  |  |  |
| *(IIIa) Ependymomas and choroid plexus tumor* | 68 | *4* | *4* | *4* | *1,1* | *1* | *1,3* | *1.9 (-1.8, 5.5)* |
| Ependymal tumors | 33 | 2 | 2 | 2 | 0,5 | 0,5 | 0,5 | 3.5 (-1.4, 8.3) |
| Choroid plexus tumors | 35 | 2 | 2 | 2 | 0,7 | 0,5 | 0,8 | 2.7 (-2.5, 7.9) |
| *(IIIb and IIId) Astrocytomas and other gliomas* | 31 | *2* | *2* | *2* | *0,5* | *0,4* | *0,5* | *-1.66 (-8.4, 5.1)* |
| Diffuse astrocytoma | - | - | - | - | - | - | - | *NA* |
| Anaplastic astrocytoma | - | - | - | - | - | - | - | *NA* |
| Unique astroctyoma variants | 27 | 2 | 2 | 2 | 0,4 | 0,4 | 0,4 | -4.4 (-13.0, 4.2) |
| Gliofibroma | <5^a^ | <1 | <1 | 0 | 0 | 0 | 0 | *NA* |
| Oligodendrogliomas | - | - | - | - | - | - | - | *NA* |
| Oligoastrocytic tumors | - | - | - | - | - | - | - | *NA* |
| Glioma , NOS | <5^a^ | <1 | 0 | <1 | 0 | 0 | 0,1 | *NA* |
| *(IIIc) Intracranial and intraspinal embryonal tumors* | - | - | - | - | - | - | - | *NA* |
| medulloblastoma, variants | - | - | - | - | - | - | - | *NA* |
| desmoplastic/nodular medulloblastoma | - | - | - | - | - | - | - | *NA* |
| PNET, variants | - | - | - | - | - | - | - | *NA* |
| medulloblastoma large cell/anaplastic | - | - | - | - | - | - | - | *NA* |
| Atypical teratoid/rhabdoid tumors | - | - | - | - | - | - | - | *NA* |
| *(IIIe) Other specified intracranial and intraspinal neoplasms* | 544 | *30* | *28* | *34* | *8,2* | *7,4* | *9,1* | ***2.7 (0.8, 4.6)*** |
| Neuronal and mixed neuronal-glial tumors | 219 | 12 | 11 | 14 | 3,4 | 2,9 | 4 | **4.9 (2.4, 7.3)** |
| tumors of the pineal region | <5^a^ | <1 | <1 | <1 | 0 | 0 | 0 | *NA* |
| Meningiomas | 43 | 2 | 3 | 2 | 0,6 | 0,8 | 0,4 | -1.08 (-6.1, 3.9) |
| tumors of the sellar region | 280 | 16 | 14 | 18 | 4,1 | 3,7 | 4,7 | 2.1 (-0.3, 4.5) |
| *(IIIf) Unspecified intracranial and intraspinal neoplasms* | 52 | *3* | *3* | *3* | *0,8* | *0,8* | *0,9* | *0.2 (-6.1, 6.4)* |
| *(Xa) Intracranial and intraspinal germ cell tumors* | - | - | - | - | - | - | - | *NA* |
| ***WHO CNS Grade*** |  |  |  |  |  |  |  |  |
| *WHO grade I* | 609 | 34 | 31 | 38 | 9,2 | 8,3 | 10,4 | ***2,8 (1.1, 4.5)*** |
| *WHO grade II* | 33 | 2 | 2 | 2 | 0,5 | 0,5 | 0,5 | *2.1 (-3.8, 8.0)* |
| *WHO grade III* | *NA* | *NA* | *NA* | *NA* | *NA* | *NA* | *NA* | *NA* |
| *WHO grade IV* | *NA* | *NA* | *NA* | *NA* | *NA* | *NA* | *NA* | *NA* |
| Unknown grade | 53 | 3 | 3 | 3 | 0,8 | 0,8 | 0,9 | *0.3 (-5.9, 6.5)* |

**Abbrevations: NA, Not Assessed -** estimation of a reliable average annual percentage change was not possible because of N = 0 in ≥1 incidence year(s)**; AAPC, Average Annual Percentage Change; 95%CI, 95 percent Confidence Interval**

^a^ Less than 5 patients are described as N<5
