# Supplementary material for: Survival rates of children and young adolescents with CNS tumors improved in the Netherlands since 1990: A population-based study
Source: Neurooncol Adv. 2021 Dec 21;4(1):vdab183. doi: 10.1093/noajnl/vdab183 (PMC9113443; doi:10.1093/noajnl/vdab183)
Supplement: vdab183_suppl_Supplementary_Table_S5 [file vdab183_suppl_supplementary_table_s5.docx]

Table S5 – Five-year observed survival and P for trend for malignant CNS tumors (excl. pilocytic astrocytomas) in children and young adolescents (aged 0-17 years) in the Netherlands

|  | **Malignant tumors (excl. pilocytic astrocytomas)** | | | | | |
| --- | --- | --- | --- | --- | --- | --- |
|  |  | **5-year OS (95%CI)** | | | |  |
|  | **N at risk** | **1990-2017** | **1990-99** | **2000-09** | **2010-17** | **P for trend^a^** |
| **Total** | 2057 | 53 (51-55) | 51 (48-55) | 47 (43-51) | 61 (57-65) | **<0.001** |
| **Sex** |  |  |  |  |  |  |
| Boys | 1198 | 54 (51-57) | 53 (48-58) | 47 (43-52) | 65 (60-70) | **<0.001** |
| Girls | 859 | 50 (47-54) | 50 (44-56) | 47 (41-53) | 56 (50-62) | **<0.001** |
| **Age at diagnosis (in years)** |  |  |  |  |  |  |
| 0 | 129 | 36 (29-46) | 34 (22-52) | 33 (22-51) | 41 (29-58) | 0.24 |
| 1-4 | 555 | 47 (43-52) | 44 (37-51) | 42 (35-49) | 57 (50-66) | **<0.001** |
| 5-9 | 601 | 50 (46-54) | 54 (47-61) | 40 (35-47) | 58 (51-66) | **<0.001** |
| 10-14 | 509 | 62 (58-67) | 56 (49-64) | 62 (55-69) | 71 (64-79) | **<0.001** |
| 15-17 | 263 | 61 (55-67) | 61 (52-72) | 52 (42-64) | 69 (60-80) | **<0.001** |
| **ICCC-3 diagnostic groups** |  |  |  |  |  |  |
| *(IIIa) Ependymomas and choroid plexus tumor* | *272* | *62 (56-68)* | *56 (47-66)* | *60 (52-71)* | *73 (62-86)* | **<0.001** |
| Ependymal tumors | 252 | 64 (58-70) | 58 (48-68) | 63 (54-73) | 76 (65-90) | **<0.001** |
| Choroid plexus tumors | 20 | 34 (19-64) | *NA* | *NA* | *NA* | *NA* |
| *(IIIb and IIId) Astrocytomas and other gliomas* | *839* | *46 (43-50)* | *56 (51-63)* | *36 (31-42)* | *48 (43-55)* | **<0.001** |
| Diffuse astrocytoma | 254 | 71 (65-77) | 68 (61-75) | 71 (60-85) | 80 (69-93) | **0.001** |
| Anaplastic astrocytoma | 68 | 22 (14-34) | 21 (9-50) | 25 (14-46) | 15 (4-50) | 0.83 |
| Unique astroctyoma variants | 20 | 75 (58-97) | *NA* | *NA* | *NA* | *NA* |
| Glioblastoma and variants | 148 | 8 (5-15) | 11 (4-32) | 8 (3-18) | 8 (3-22) | 0.57 |
| Oligodendrogliomas | 58 | 69 (58-82) | 67 (52-85) | 61 (42-88) | 100 | 0.11 |
| Oligoastrocytic tumors | 26 | 58 (42-80) | *NA* | *NA* | *NA* | *NA* |
| Glioma, NOS | 265 | 42 (36-48) | 37 (20-66) | 31 (24-41) | 52 (44-61) | 0.18 |
| *(IIIc) Intracranial and intraspinal embryonal tumors* | *616* | *52 (48-56)* | *48 (42-56)* | *48 (42-54)* | *62 (55-70)* | **<0.001** |
| medulloblastoma, variants | 377 | 59 (54-64) | 56 (48-66) | 51 (43-60) | 74 (65-84) | **<0.001** |
| desmoplastic/nodular medulloblastoma | 68 | 67 (56-80) | 44 (25-76) | 70 (53-93) | 79 (65-96) | 0.17 |
| PNET, variants | 106 | 33 (25-43) | 33 (22-50) | 34 (23-51) | 25 (10-62) | 0.43 |
| medulloblastoma large cell/anaplastic | 14 | 43 (22-86) | *NA* | *NA* | *NA* | *NA* |
| Atypical teratoid/rhabdoid tumour | 51 | 24 (15-40) | 0 | 33 (18-61) | 20 (9-47) | 0.69 |
| *(IIIe) Other specified intracranial and intraspinal neoplasms* | *43* | *50 (37-68)* | *33 (13-84)* | *44 (25-76)* | *66 (47-92)* | 0.41 |
| Neuronal and mixed neuronal-glial tumors | 12 | 62 (38-100) | *NA* | *NA* | *NA* | *NA* |
| tumors of the pineal region | 23 | 48 (31-73) | *NA* | *NA* | *NA* | *NA* |
| Meningiomas | 8 | *NA* | *NA* | *NA* | *NA* | *NA* |
| tumors of the sellar region | - | - | - | - | - | *NA* |
| *(IIIf) Unspecified intracranial and intraspinal neoplasms* | *161* | *55 (47-63)* | *27 (18-39)* | *55 (40-77)* | *86 (77-95)* | **<0.001** |
| *(Xa) Intracranial and intraspinal germ cell tumors* | *126* | *78 (71-85)* | *67 (55-81)* | *85 (75-97)* | *85 (74-98)* | **<0.001** |
| ***WHO CNS Grade*** |  |  |  |  |  |  |
| *WHO grade I* | *21* | *100* | *NA* | *NA* | *NA* | *NA* |
| *WHO grade II* | *448* | *76 (72-80)* | *73 (67-79)* | *75 (67-83)* | *86 (79-94)* | **<0.001** |
| *WHO grade III* | *275* | *39 (34-45)* | *31 (22-43)* | *39 (31-48)* | *48 (38-61)* | **<0.001** |
| *WHO grade IV* | *805* | *43 (40-47)* | *41 (35-48)* | *39 (34-45)* | *50 (44-57)* | **<0.001** |
| *Unknown grade* | *382* | *45 (40-50)* | *29 (21-41)* | *32 (25-41)* | *63 (56-70)* | **<0.001** |

**Abbrevations: NA, Not Assessed** – due to the low number of cases**; OS, Observed Survival; 95%CI, 95 percent Confidence Interval**

^a^ Survival changes over time were evaluated by using Poisson regression modelling adjusted for follow-up time (in years) in which the variable period of diagnosis was entered as a continuous variable in the model
