# Supplementary material for: Survival rates of children and young adolescents with CNS tumors improved in the Netherlands since 1990: A population-based study
Source: Neurooncol Adv. 2021 Dec 21;4(1):vdab183. doi: 10.1093/noajnl/vdab183 (PMC9113443; doi:10.1093/noajnl/vdab183)
Supplement: vdab183_suppl_Supplementary_Table_S6 [file vdab183_suppl_supplementary_table_s6.docx]

Table S6 – Five-year observed survival and P for trend for pilocytic astrocytomas in children and young adolescents (aged 0-17 years) in the Netherlands

|  | **Pilocytic astrocytomas (9421/1, 9421/3, 9425/3^b^)** | | | | | |
| --- | --- | --- | --- | --- | --- | --- |
|  |  | **5-year OS (95%CI)** | | | |  |
|  | **N at risk** | **1990-2017** | **1990-99** | **2000-09** | **2010-17** | **P for trend^a^** |
| **Total** | 885 | 95 (94-97) | 94 (91-97) | 95 (93-97) | 98 (96-100) | **<0.001** |
| **Sex** |  |  |  |  |  |  |
| Boys | 429 | 97 (95-99) | 97 (94-100) | 96 (93-99) | 99 (98-100) | **<0.001** |
| Girls | 456 | 94 (92-96) | 91 (86 - 96) | 94 (91-98) | 96 (93-100) | **<0.001** |
| **Age at diagnosis (in years)** |  |  |  |  |  |  |
| 0 | 29 | 64 (49-85) | 75 (43-100) | 55 (32-94) | 70 (49-100) | *NA* |
| 1-4 | 236 | 96 (93-98) | 90 (83-97) | 98 (95-100) | 98 (95-100) | **<0.001** |
| 5-9 | 299 | 97 (95-99) | 95 (91-100) | 97 (94-100) | 100 | **<0.001** |
| 10-14 | 207 | 96 (93-99) | 97 (92-100) | 94 (90-99) | 98 (95-100) | **0.004** |
| 15-17 | 114 | 97 (94-100) | 96 (90-100) | 94 (86 - 100) | 100 | **0.01** |

**Abbrevations: NA, Not Assessed** – due to the low number of cases**; OS, Observed Survival; 95%CI, 95 percent Confidence Interval**

^a^ Survival changes over time were evaluated by using Poisson regression modelling adjusted for follow-up time (in years) in which the variable period of diagnosis was entered as a continuous variable in the model

^b^ Pilomyxoid astrocytoma n=14, between 2005 and 2016
