# Supplementary material for: Survival rates of children and young adolescents with CNS tumors improved in the Netherlands since 1990: A population-based study
Source: Neurooncol Adv. 2021 Dec 21;4(1):vdab183. doi: 10.1093/noajnl/vdab183 (PMC9113443; doi:10.1093/noajnl/vdab183)
Supplement: vdab183_suppl_Supplementary_Table_S7 [file vdab183_suppl_supplementary_table_s7.docx]

Table S7 – Five-year observed survival and P for trend for non-malignant CNS tumors (excl. pilocytic astrocytomas) in children and young adolescents (aged 0-17 years) in the Netherlands

|  | **Non-Malignant tumors (excl. pilocytic astrocytomas)** | | | | |
| --- | --- | --- | --- | --- | --- |
|  |  | **5-year OS (95%CI)** | | |  |
|  | **N at risk** | **2000-17** | **2000-09** | **2010-17** | **P for trend^a^** |
| **Total** | 695 | 99 (98-100) | 98 (97-100) | 100 (99-100) | **<0.001** |
| **Sex** |  |  |  |  |  |
| Boys | 307 | 99 (97-100) | 98 (95-100) | 100 | **<0.001** |
| Girls | 388 | 99 (98-100) | 99 (98-100) | 100 (99-100) | **<0.001** |
| **Age at diagnosis (in years)** |  |  |  |  |  |
| 0 | 25 | 92 (82-100) | 87 (71-100) | 100 | *NA* |
| 1-4 | 102 | 98 (95-100) | 98 (95-100) | 98 (94-100) | **0.006** |
| 5-9 | 139 | 99 (98-100) | 99 (96-100) | 100 | 0.07 |
| 10-14 | 207 | 100 (99-100) | 99 (97-100) | 100 | **0.008** |
| 15-17 | 222 | 99 (98-100) | 99 (97-100) | 100 | **0.02** |
| **ICCC-3 diagnostic groups** |  |  |  |  |  |
| *(IIIa) Ependymomas and choroid plexus tumor* | *68* | *99 (96-100)* | *100* | *97 (91-100)* | *1.00* |
| Ependymal tumors | 33 | 97 (91-100) | 100 | 93 (80-100) | *1.00* |
| Choroid plexus tumors | 35 | 100 | 100 | 100 | *1.00* |
| *(IIIb and IIId) Astrocytomas and other gliomas* | *31* | *100* | *100* | *100* | *1.00* |
| Diffuse astrocytoma | - | - | - | - | *NA* |
| Anaplastic astrocytoma | - | - | - | - | *NA* |
| Unique astroctyoma variants | 27 | 100 | 100 | 100 | *1.00* |
| Gliofibroma | <5^b^ | *NA* | *NA* | *NA* | *NA* |
| Oligodendrogliomas | - | - | - | - | *NA* |
| Oligoastrocytic tumors | - | - | - | - | *NA* |
| Glioma, NOS | <5^b^ | *NA* | *NA* | *NA* | *NA* |
| *(IIIc) Intracranial and intraspinal embryonal tumors* | - | - | - | - | *NA* |
| medulloblastoma, variants | - | - | - | - | *NA* |
| desmoplastic/nodular medulloblastoma | - | - | - | - | *NA* |
| PNET, variants | - | - | - | - | *NA* |
| medulloblastoma large cell/anaplastic | - | - | - | - | *NA* |
| Atypical teratoid/rhabdoid tumour | - | - | - | - | *NA* |
| *(IIIe) Other specified intracranial and intraspinal neoplasms* | *544* | *99 (98-100)* | *98 (96-100)* | *100* | ***<0.001*** |
| Neuronal and mixed neuronal-glial tumors | 219 | 98 (96-100) | 96 (93-100) | 100 | ***<0.001*** |
| tumors of the pineal region | <5^b^ | *NA* | *NA* | *NA* | *NA* |
| Meningiomas | 43 | 100 | 100 | 100 | *1.00* |
| tumors of the sellar region | 280 | 99 (98-100) | 99 (97-100) | 100 | **0.01** |
| *(IIIf) Unspecified intracranial and intraspinal neoplasms* | *52* | *100* | *100* | *100* | *1.00* |
| *(Xa) Intracranial and intraspinal germ cell tumors* | - | - | - | - | *NA* |
| ***WHO CNS Grade*** |  |  |  |  |  |
| *WHO grade I* | 609 | 99 (98-100) | 98 (97-100) | 100 (99-100) | **<0.001** |
| *WHO grade II* | 33 | 97 (91-100) | 94 (84-100) | 100 | 0.08 |
| *WHO grade III* | *NA* | *NA* | *NA* | *NA* | *NA* |
| *WHO grade IV* | *NA* | *NA* | *NA* | *NA* | *NA* |
| *Unknown grade* | 53 | 100 | 100 | 100 | *0.541* |

**Abbrevations: NA, Not Assessed** – due to the low number of cases**; OS, Observed Survival; 95%CI, 95 percent Confidence Interval**

^a^ Survival changes over time were evaluated by using Poisson regression modelling adjusted for follow-up time (in years) in which the variable period of diagnosis was entered as a continuous variable in the model

^b^ Less than 5 patients are described as N<5
