# Supplementary material for: Survival rates of children and young adolescents with CNS tumors improved in the Netherlands since 1990: A population-based study
Source: Neurooncol Adv. 2021 Dec 21;4(1):vdab183. doi: 10.1093/noajnl/vdab183 (PMC9113443; doi:10.1093/noajnl/vdab183)
Supplement: vdab183_suppl_Supplementary_Table_S8 [file vdab183_suppl_supplementary_table_s8.docx]

Table S8 - Mortality from CNS tumors (ICD-10 C70-C72) between 1970-2017 in children and young adolescents (aged 0-19 years) in the Netherlands

|  |  | **1970-2017** | **1970-79** | **1980-89** | **1990-99** | **2000-10** | **2010-17** | **AAPC (95%CI) 1970-2017** |
| --- | --- | --- | --- | --- | --- | --- | --- | --- |
| ***Total*** | *Average number of deaths/ year* | 41 | 56 | 41 | 35 | 38 | 34 |  |
|  | *Age standardized mortality rate (per 1 million)* | 10.2 | 12.8 | 10.2 | 9.2 | 9.7 | 8.8 | **-0.9 (-1.3,-0.4)** |
|  |  |  |  |  |  |  |  |  |
| ***Boys*** | *Average number of deaths/ year* | 23 | 33 | 24 | 19 | 22 | 17 |  |
|  | *Age standardized mortality rate (per 1 million)* | 11.1 | 14.6 | 11.5 | 9.6 | 10.8 | 8.7 | **-1.1 (-1.7,-0.6)** |
| ***Girls*** | *Average number of deaths/ year* | 18 | 23 | 17 | 16 | 16 | 16 |  |
|  | *Age standardized mortality rate (per 1 million)* | 9.2 | 10.9 | 8.8 | 9 | 9 | 8.9 | **-0.6 (-1.2, -0.0)** |
|  |  |  |  |  |  |  |  |  |
| ***0-1*** | *Average number of deaths/ year* | 2 | 3 | 2 | 2 | 1 | 1 |  |
|  | *Age specific mortality rate (per 1 million)* | 9.4 | 17 | 8.9 | 7.7 | 7.2 | 5.6 | **NA** |
| ***1-4*** | *Average number of deaths/ year* | 10 | 16 | 9 | 8 | 9 | 7 |  |
|  | *Age specific mortality rate (per 1 million)* | 12.6 | 18.6 | 12.7 | 10.8 | 11 | 9.2 | -**1.6 (-2.5,-0.7)** |
| ***5-9*** | *Average number of deaths/ year* | 12 | 14 | 11 | 9 | 13 | 12 |  |
|  | *Age specific mortality rate (per 1 million)* | 11.6 | 12.0 | 11.6 | 9.2 | 13.2 | 12.2 | 0.2 (-0.5,1.0) |
| ***10-14*** | *Average number of deaths/ year* | 9 | 13 | 9 | 7 | 8 | 9 |  |
|  | *Age specific mortality rate (per 1 million)* | 8.6 | 10.3 | 8.2 | 8.1 | 7.6 | 8.9 | -0.5 (-1.4,0.5) |
| ***15-19*** | *Average number of deaths/ year* | 8 | 10 | 10 | 9 | 7 | 5 |  |
|  | *Age specific mortality rate (per 1 million)* | 7.8 | 8.8 | 8.3 | 9.1 | 7.2 | 5.3 | **-1.2 (-2.0,-0.3)** |

**Abbrevations: AAPC, Average Annual Percentage Change; NA, Not Assessed**  estimation of a reliable average annual percentage change was not possible because of N = 0 in ≥1 incidence year(s)**; 95%CI, 95 percent Confidence Interval**
